# College Students and Eating Habits: A Study Using An Ecological Model for Healthy Behavior

**DOI:** 10.3390/nu10121823

**Published:** 2018-11-23

**Authors:** Giovanni Sogari, Catalina Velez-Argumedo, Miguel I. Gómez, Cristina Mora

**Affiliations:** 1 Department of Food and Drug, University of Parma, 43124 Parma, Italy; cristina.mora@unipr.it; 2 Charles H. Dyson School of Applied Economics and Management, Cornell University, Ithaca, NY 14850, USA; mig7@cornell.edu; 3 Tecnológico de Monterrey, EGADE Business School, San Pedro Garza García 66269, Mexico; catalina.velez@gmail.com

**Keywords:** young adults, focus group, USA, interventions, overweight, qualitative studies

## Abstract

Overweightness and obesity rates have increased dramatically over the past few decades and they represent a health epidemic in the United States (US). Unhealthy dietary habits are among the factors that can have adverse effects on weight status in young adulthood. The purpose of this explorative study was to use a qualitative research design to analyze the factors (barriers and enablers) that US college students perceived as influencing healthy eating behaviors. A group of Cornell University students (*n* = 35) participated in six semi-structured focus groups. A qualitative software, CAQDAS Nvivo11 Plus, was used to create codes that categorized the group discussions while using an Ecological Model. Common barriers to healthy eating were time constraints, unhealthy snacking, convenience high-calorie food, stress, high prices of healthy food, and easy access to junk food. Conversely, enablers to healthy behavior were improved food knowledge and education, meal planning, involvement in food preparation, and being physically active. Parental food behavior and friends’ social pressure were considered to have both positive and negative influences on individual eating habits. The study highlighted the importance of consulting college students when developing healthy eating interventions across the campus (e.g., labeling healthy food options and information campaigns) and considering individual-level factors and socio-ecological aspects in the analysis.

## 1. Introduction

Overweightness and obesity rates have dramatically increased over the past few decades and they represent a health epidemic in the United States, as well as in many other areas of the world [1,2,3]. According to a scoping review of risk behavior interventions in young men, Ashton, Hutchesson, Rollo, Morgan & Collins [4] identified obesity as a serious health risk with an incidence rate of obesity reaching 29% of the population aged 20–39 years old [5,6]. Physical inactivity and unhealthy dietary habits are among the main behaviors that potentially have adverse effects on weight status in young adulthood, and consequently, the future health of adults [3,7].

As reported by the World Health Organization (WHO) [8], the adult disease burden is due to health risk behaviors that start during adolescence (e.g., unhealthy eating practices). For example, most of the United States (US) population does not consume the recommended daily amount of fruit and vegetables, nuts, and seeds. On the other hand, the consumption of added sugars, processed meats, and trans fats is higher than the recommended daily intake [9]. It has been shown that after the transition from adolescence to young adulthood, when independency increases, young adults are continuously challenged to make healthful food choices [2,10]. Along with unhealthy eating behaviors, a new series of weight-related behavioral patterns begins throughout this period, such as excessive alcohol consumption and a low level of physical activity.

Substantial life-changing transitions happened when young adults finish high school to start college or a working life [10]. According to the literature [11,12,13], university is a critical period for young adults regarding food choices and their relationship with weight gain. Some studies have even shown that college students tend to gain more weight than those who do not attend university [14]. In order to design and support healthy nutrition campaigns (e.g., less meat options) across campuses, it is critical to improve knowledge of dietary behaviors in the university-age population [15].

In the last decades, there has been growing interest in the development and implementation of health promotion interventions in the workplace [16]. Studies exploring eating behavior in children [17], adolescents [18,19], and young adults [20] have been done in recent years; however, theories to explain such behaviors are still moving from the nascent to the mature stage [21].

Recently, the so-called Ecological Model has been considered as an acceptable framework to link individual and social behaviors with environmental determinants, to reduce serious and prevalent health problems [22].

The aim of this study is to explore the barriers and enablers of healthy eating behaviors among US college students, using focus groups that foster open discussion between a small number of participants. This study is the first stage of a larger research project called “CONSUMEHealth. Using consumer science to improve healthy eating habits”, funded bythe European Union’s Horizon 2020 research and Innovation programme (Marie Sklodowska-Curie grant agreement No 749514).

## 2. Materials and Methods

### 2.1. Focus Groups

We selected focus group interviewing as a key methodology for the study, the elements of which include participant observation, formal and informal interviewing, filming, and recording, among others [23]. Focus groups are used to obtain insights and in-depth information on why and how people think (perceptions, attitude, opinions, experience) about a topic of interest [24] used to unlock the complexity of the decision-making process [25] and unencumbered by what we expect to find [26]. In our study, a focus group was suitable, since we were aiming to obtain cultural insights from a group of individuals, and to explore their beliefs and behaviors [27], allowing for us to examine the context of healthy eating behaviors [16,28]. Various studies have demonstrated that focus groups are an appropriate research method to study eating habits, particularly among students [2,29]. Since the definition of this population was not just a matter of age, but of lifestyle and identity, a focus group could help us to better understand the meanings of healthy eating behaviors and its contexts.

### 2.2. Participants

Eligible participants were college students aged 18 to 25 years, who were transitioning from adolescence to young adulthood, who lived in the USA, and who were enrolled at Cornell University in the town of Ithaca (New York, NY, USA). Similar to previous studies [29], no first-year university students were included in the study, due to their limited college experience. In addition, we excluded students from nutrition classes or any other disciplines that might transmit a greater overall knowledge or awareness of healthy eating. The final group consisted of students from different disciplines (humanistic and scientific). These young adults were recruited via flyers that were distributed across the University facilities, and via email using a college student database. In the advertisement sheet, a link to an online survey was provided to facilitate recruitment, and to give subjects the essential statement outline of the study (aim, benefits, and risks that are associated with time, incentives, other). One advantage of our approach was that it allowed us to recruit participants from different disciplines and years of study. In addition, we chose to have mixed-gender groups, which could produce a greater variety of responses and better discussion [29]. The interview guideline was designed to take participants on a journey, starting from a broader concept of health, to more specific questions on past, present, and future diet behavior practices.

### 2.3. Procedure

The recruitment of participants was carried out using an online system at Cornell University. A recruitment rate of between six and eight participants per focus group session was planned, in order to have at least four people in each focus group session, therefore, an over-recruitment of two students was planned in the case of ‘no-shows’.

Following the literature [29], a semi-structured question guide was developed to identify the key questions for the research problem (eating habits, physical activity levels, and weight change). Enough flexibility and side-questions allowed for open discussions within the group, to obtain more in-depth information from participants.

Projective techniques were used both at the beginning of the sessions for “ice-breaking”, and later on to understand better emotional connections and cognitions towards the topic of interest [30]. Specifically, the photograph response test technique was used, which consists of showing a series of photographs that are related to the topic under investigation. A stimulus (images of obese/overweight individuals) was presented to the group, and the participants were asked to answer with the first words that came to their mind.

As reported by Guerrero and Xicola [24], the integration of different qualitative techniques (e.g., projective stimuli as in this study) within the same focus group was considered to be a mixed approach. The study was approved by the Institutional Review Board (IRB) of the Office of Research Integrity and Assurance of Cornell University (Protocol ID: 1709007406).

### 2.4. Data Collection Outline

During the online prescreening registration, all of the participants completed a short questionnaire, providing self-reported socio-demographic information, physical activity, height, weight status, and perceived body image.

Before beginning the focus group, an information sheet about the study and a consent form for anonymity and confidentiality were signed by each participant. Drinks and a few snacks were provided in order to make the environment as much comfortable as possible. In addition, the room that was used to carry out the discussions was modified to look like a living room of a house.

As suggested in the literature [28], each focus group lasted around 90 min, and it was held in a comfortable and quiet place. The sessions were video-audio recorded with the permission of the participants, and were facilitated by a well-trained and experienced moderator (female moderator with five years of experience in focus groups in the field of food, both in the public and private context). The principal investigator was an observer, and stayed in another room that was connected with audio and video recording systems during the focus group discussion. The moderator directed the flow of the discussion, and ensured that all of the important issues were covered. We opted for small groups (4–6 people), which was considered to be more appropriate when the topic of investigation is seen as complex and personal [31]. Both the principal investigator and the moderator did not have any type of relationship with the participants; we strongly believe that no bias or conflict of interest exist between the research team, the subjects, and the focus of the study.

The semi-structured questions guide (Table 1), as developed following Krueger and Casey [27], aimed to investigate the main factors influencing eating behaviors among college students. First, a projective technique was first used for “ice breaking”, and to facilitate the group discussion. At the beginning, all of the participants were asked to list “five healthy eating habits” and “five unhealthy eating habits”, and afterwards to read the list out and share it with group. In this way, the whole group was actively involved in the discussion, and participants became acquainted with, and felt connected with each other. The main questions focused on factors influencing students’ health and weight-related behaviors. Before ending each of the focus group sessions, the moderator and principal investigator decided whether further questions were needed. At the very end of the focus group, all of the subjects chose to either receive a monetary payment ($ 15) or university course credit (1.5) for their participation.

### 2.5. Data Analysis

In the field of health studies, the use of focus groups for research is a relatively recent phenomena [28]. The information resulting from focus groups is usually analyzed throughout a process of categorizing and coding the data in a systematic manner.

At the end of the six focus group sessions, the audio tapes were transcribed verbatim in Microsoft Word by an independent transcription agency, and they were double-reviewed by two researchers. Second, the data collected were analyzed by the principal investigator and two research assistants who were trained in qualitative analysis. All quotes were encoded using the computer-assisted qualitative data analysis software Nvivo11 Plus Version 11 (QSR International Pty Ltd., Melbourne, Australia) [32]. This software helped the researchers at the stage of data analysis, marking, and coding the transcription, and helped them to identify the relations between categories (concepts, themes, and ideas) and individuals [28].

An inductive thematic approach, which is useful for identifying core meanings that were relevant to the research objects, was used for data analysis, in which quotes were coded and categorized into themes and subthemes [25,33]. These themes were organized into individual, social, and environmental categories using an Ecological Model framework [16,22], and were successively described. A Microsoft Excel package was used to analyze the characteristics of the sample using responses from the questionnaire (descriptive statistics).

## 3. Results

### 3.1. Descriptive Results

In our study, six focus group discussions were conducted until saturation of new information was reached. The final sample consisted of 35 students (23 females), with a mean age of 20.4 ± 1.5 years and a mean body mass index (BMI) of 23.2 (SD ± 4.52), which was calculated as weight (kg) divided by height squared (m^2^). Most participants considered themselves to have a healthy weight status, and few of them indicated current or past eating disorders. The characteristics of the sample are summarized in Table 2. Participants were also from a variety of study disciplines and different college years (from junior to senior). This variety in participant characteristics enormously contributed to gather more insights (e.g., diverse experiences and opinions) into the relationship between behaviors and healthy eating.

### 3.2. Qualitative Results

Following the literature [31], the researchers reviewed the transcript line-by-line encoding and classified the text. As a first step, the questions that were enclosed in the script were used as initial categories, then during a rigorous and systematic reading of the transcript, the main categories started to emerge [33]. The researchers used an inductive coding method to find meaningful messages to categorize into main themes and sub-themes.

The information was then analyzed in conjunction with the Ecological Model conceptual framework. The importance of the Ecological Model in the social sciences is the consideration of interactions between the people’s behavior and the environment (sociocultural, policy, and physical) [16,29].

**Figure 1:**
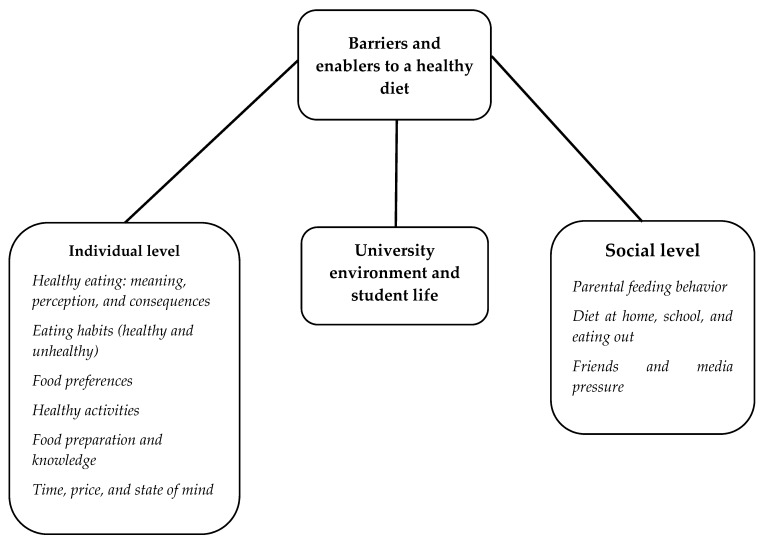
Factors influencing healthy eating behaviors of college students.

With the results from this model, we developed a list of factors influencing healthy eating behaviors among college students, based on content analysis of the focus groups (Figure 1). We adapted a model by Deliens, Clarys, Bourdeaudhuij & Deforche [29], and then developed the following main levels for the analysis: individual (intrapersonal), social (interpersonal relationship), and university environment (community settings), and some main attributes of the students (e.g., gender). The most significant quotes by respondents were reported to illustrate each (sub)theme. We also decided to incorporate some basic information of the participants by using an ID for the quotes: e.g., FG1_F21 (Focus Group 1, Female, age 21 years old).

#### 3.2.1. Individual Level (Intrapersonal)

Intrapersonal factors are represented mainly by attitude, behavior, self-concepts, and skills [16].

##### Healthy Eating: Meaning, Perception, and Consequences

Research shows that individuals’ beliefs about a healthy diet is shaped by their psychology. Understanding what healthy eating means is crucial to making healthy food choices across and within product categories. Participants seemed to be aware of healthy eating habits: “*For me, healthy eating is eating clean. So, lots of fresh veggies and fruits and some sort of protein*” (FG1_F20); however, they were also aware that they did not necessarily follow this suggestion: “*Things* (healthy food) *that help fulfil your daily nutrition requirement, even though I obviously don’t do that*” (FG1_F20).

There was a gap between having knowledge and actually practicing it: “*… now I feel like I’m more aware of it* (healthy eating), *I just don’t pay attention to it*” (FG5_F21). In addition, they highlighted how the meaning of healthy eating had changed over the past decades: “*when I was a kid, I definitely thought it was more ... just eating less, ... now I understand that it’s more eating the right things, and not necessarily eating less, but just eating different stuff*”(FG1_F21).

During the focus groups, the term “healthy” itself proved to be quite elastic: “*I think about getting a lot of balance*” (FG3_M23) and it was perceived to have changed overtime: “*before, it was all about portion control, eating smaller things, but now, it’s focused more on eating healthy things*” (FG1_F20). Most participants considered their generation to be more health-aware and more health-conscious than the previous ones. However, others believed that today, it is harder for people to eat healthy because there is so much fast food available. For someone whose parents taught them during childhood, healthy eating remained an important factor for the future: “*my mom told me when I was a kid, healthy eating is if your plate is colourful, so sometimes when I went through that little phase where I was trying to eat really well at the dining halls I’d be like, carrots, orange, tomatoes, red, I’d get a bowl of blueberries, blue. You’d try to get every colour on your plate and that’s healthy*” (FG5_F19).

Participants were aware of the long-run consequences of not maintaining a healthy diet: “*It’s risk for diseases, increasing your risk of dying earlier*” (FG4_F19); “*you have less health problems, for the most part, that are related to your diet. You probably have more energy, honestly, because processed stuff sort of slows you down*” (FG1_F20). In particular, a male participant reported: “*I think that America has this epidemic, which is obesity. And I know that leads to a whole bunch of complications, especially the demographic that I am. I understand that our life expectancy isn’t as high as other demographics, and that’s due to obesity, diabetes, heart disease and stuff like that*” (FG2_M20).

They also considered “eating healthy” as something that was related to a lifestyle with positive consequences to the general mindset of the individual: “*I think healthy is feeling good about yourself, having energy, and not being exhausted all day*” (FG2_F18); “*I think healthy goes beyond just food, you have to be mentally healthy and physically healthy*” (FG2_F19); “*I tend to like healthy food, it makes me feel better*” (FG6_M22). More generally, people related the concept of being healthy to both physical and psychological status: “*I think being healthy is both your physical appearance and your mindset… exercised and eating food, as well as balancing it out with your mental state*” (F2_M20).

We used a projective technique to create more interaction and interest on the topic. Images of overweight/obese people were shown, and participants were then asked what thoughts came into their mind. Most participants felt uncomfortable with describing these images. Some of them thought that being heavily overweight or obese could be attributed to not having control over their own lifestyle: “*I feel bad for them, because I know the probably inside, they are not happy with themselves, but it’s all your personal choice*” (FG3_M19). At the same time, there was a feeling both of sadness for them, but also a willingness to not judge other people’s weight status. Only one person mentioned that body image was a motivator in maintaining healthy eating: “*I want to be in a good shape, and I think that’s what motivates me*” (FG4_M21).

##### Eating Habits (Healthy and Unhealthy)

Every participant was asked to list five healthy and five unhealthy eating habits on post-it notes and then share it among the groups (Table 3). First, snacking was associated most of the time with unhealthy eating, as mentioned by several participants: “*I’m trying to eat a heavier breakfast so that I snack less throughout the day*” (FG1_F21); “*I have snacks late night, mostly, if I’m going to snack at all, it’s generally junk food*” (FG4_M19). Only a few of them tried snacking with an healthy option: “*I don’t mindlessly snack, but when I do snack, it’s always something healthy like nuts or fruit*” (FG4_F19). Some participants did not seem conscious of having three meals a day, but preferred to have smaller snacks consistently throughout the day and being portion-aware: “*I try to eat like four to five times a day like smaller meals as opposed to just like breakfast, lunch and dinner*” (FG3_M23). Regarding drinking habits, surprisingly, alcohol consumption was not mentioned as an unhealthy drinking habit; but more attention was focused on the most common daily drinks (i.e., water, coffee, and soda). One female participant said: “*I like carbonated drinks, like sugary drinks that I should probably stay away from*” (FG1_F21). Many people were aware that a high sugar-sweetened beverage intake was associated with greater weight gain.

The participants were asked about why American consumers do not follow the dietary guidelines given by the United States Department of Agriculture (USDA). Most of them mentioned that nowadays there is a greater availability of unhealthy foods: “*I think there’s a lot more junk food now than there was then, and it’s also way cheaper than getting healthy food*” (FG1_F20); “*I think junk food is way more accessible than going out to get healthy food*” (FG1_F21); “*sometimes people just don’t have access to food in their neighbourhood*” (FG6_M22).

##### Food Preferences

Food preferences are highly complex, personal, and influenced by a broad variety of factors, especially physiological. Even if health seemed to be important for everyone, when choosing food, students did not take health into consideration as the most important factor, but usually pleasure and taste. As one participant said: “*I think unhealthy food just tastes better. I don’t know, if a food tastes good to me, I have thoughts of, "Is this unhealthy?" Because I feel like healthy food just doesn’t taste as good*” (FG2_F19). Likeability as a first factor for choosing food was confirmed by another student: “*I think unhealthier food just tastes better to everybody*” (FG2_M20). Another participant highlighted the importance of the pleasure of eating: “*I really like pasta, like a lot, it’s pretty much what I eat every day. I put hot sauce on everything*” (FG5_F19).

##### Healthy Activities

Almost all of the participants mentioned that they had been very busy since they started tertiary education, and that this was a barrier to maintaining a healthy lifestyle. They remembered that exercising was as a big part of family time: “…*me and my two brothers and my dad, we started going to the gym. So we’d go to the gym like every weekend*” (FG2_M20); “*I play a lot of soccer with my dad*” (FG3_M19). It is clear the role of parents in incentiving activities to stay healthy: “*my parents were also very encouraging of me and my other siblings with doing sports*” (FG6_M21). Nowadays, due to time constraints associated with being a college student, it was more difficult to stay active. The statement “*not keeping junk food in the house*” was repeated by several students as a way to avoid the temptation of eating unhealthy foods, as was having small snacks throughout the day rather than designated meals. They were also aware about overeating, and few of them believed themselves to be good at controlling portion sizes: “*I try to get individual packages, so I have portion control*” (FG2_F18).

##### Food Preparation and Knowledge

In order to eat healthy, consumers must have some knowledge about food, healthful products, and the composition of a meal, among others. During the focus groups, participants were asked about changes that they had made in their cooking habits since they had moved from home. Some of them realized how negative the changes were in terms of eating healthy: “*the first time I lived outside of home wasn’t good. I ate out twice a day, every day, which is really unhealthy and really expensive. So now I’m trying to cook more, which is good. I feel like I’m healthier when I’m cooking it myself*” (FG1_F21). Others confirmed how expensive it is to eat out frequently: “*Well I didn’t cook at all when I was at home. So just off campus, it’s cheaper to cook than eating out every night, so I’m just trying to cook more*” (FG1_F21).

Students were asked their involvement in preparing food when living with their parents, the majority declared to have never helped in the kitchen or only during holiday meals. One participant shared a personal experience: “*Only for Thanksgiving or Christmas I would usually make a dessert or something like that. Cake or cookies*” (FG6_M21).

When asked to elaborate more on a healthy diet and give examples, few students had a vague idea of what the Mediterranean diet was about: “*I’ve definitely heard of it before, but I don’t ... is it like, only eating certain Greek, Mediterranean ingredients?*” (FG2_F19), and most of them had not even heard of the term before.

##### Time, Price and State of Mind

The transition from living at home to the college experience was considered to be stressful. Most of the participants mentioned a problem with stress eating, especially when studying; as one participant said: “*… I definitely snack too much when I’m stressed*” (FG4_F19). Another one: “*I work too much. I don’t take the down time to exercise. I like to snack a lot. I use food to regulate my mood*” (FG6_M22). Almost all participants believed that they did not have enough time to prepare healthy meals. The “lack of time” appeared to be an important barrier: “*I don’t have time to be going to the grocery store to just get fruit and healthy things*” (FG1_F20). Time constraints also made students skip meals: “*…then sometimes I will eat at random hours during the day, including sometimes I’ll have to skip lunch if I just don’t have enough time, which I can see the effects, it just makes me really tired, it’s not good for working out*” (FG4_F19).

Also, the relative perception of the high costs of buying healthy food (i.e., fruits and vegetables) was one of the main barriers to a varied diet [2,34]. For many students: “*junk food is way cheaper than getting healthy food*”; as one female participant specified: “*it can be hard to afford healthy food, because no matter what healthy eaters say about how easy is to find cheap, healthy food, it’s always probably gonna be cheaper to find heavily processed junk food*” (FG1_F20). Another female participant with Asian origin confirmed with her personal experience that: “*it’s very abnormal in America that the fruit and the vegetables are much expensive than the meat, because back in China the vegetables and fruits are very cheap, so everyone can have access to that*” (FG5_F24).

#### 3.2.2. Social Level (Interpersonal Relationships)

Social relationships in early adulthood are predominantly formed with roommates and friends at college, as well as with family members, even if with a lower frequency with the latter. The perception of social pressure was a strong determinant in supporting and maintaining a healthy diet [35]. As one participant said in relation to healthy eating: “*What you eat and who you’re around is really influential*” (FG2_F20). Another one confirmed this point: “*Seeing if someone’s eating really unhealthy, you can be like: "I’m going to be the one to eat healthy tonight", or if everyone’s eating healthy, you feel more inclined to eat healthy*” (FG2_F20). Sometimes, it was also the influence of the partner that could make a person change their dietary habits.

##### Parental Feeding Behavior

Respondents were asked about how parents can negatively and positively impact a child’s eating behavior. They agreed that it was difficult for kids and adolescents to learn about eating healthy if their parents did not influence and teach them: “*I think as a child, you look up to your parents a lot, so instead of verbally saying, "Eat healthy, blah blah blah…" you actually have to show it*” (FG2_F19).

One student explained that sometimes there was a risk that the parents were too busy to take care of their children’s diet: “*If parents are too busy or they don’t have the income and also the time, if they’re working too many jobs, you know, they’ll just get packaged food or processed foods and that could definitely have a very negative effect*” (FG6_M21). As a result, the parents prefer to give them money to buy food away from home and most of them choose junk food or fast food: “*if I’m with my friends, I can kind of get away with my mom not knowing what I’m eating. So I tend to eat what I can’t eat at home, so always unhealthy*” (FG1_F21).

These young adults believed that parents should give a good example (i.e., not going to a fast food place). Most of the students mentioned the role of the mother as a relevant figure for giving good recommendations: “*my mom has always ingrained the healthy eating thing in me*” (FG1_F20); “*when I was younger ... even now, my mom only has healthy food available for me. And if I ever shop with her, she doesn’t let me buy snacks or sweets*” (FG1_F21). The participants who mentioned that their parents were good at cooking, and liked preparing foods from different cultures, also realized that they should not be really picky in their food choices. Others reported that their parents used some tricks to make their children to eat healthy food: “*I think my parents just seasoned my vegetables so it would taste better. And that way I wouldn’t really have to think about me eating vegetables*” (FG4_M20). Other students experienced a more ambiguous and controversial approach with food: “*We weren’t allowed to leave the table until I finished my food*” (FG4_F21); in this case, sometimes their mothers were part of the "Clean Plate Club”, a club where parents are used to asking their children to finish everything on their plates.

##### Dietary Aspects of Home, School, and Eating Out

Respondents were asked what different eating behaviors they had between eating out and at home. Even if young adults ate in a variety of different settings, especially after living with their parents, the number of times eating out strongly increased. For instance, eating at home was usually correlated with higher fruit and vegetable intakes. However, many participants said that eating out was a kind of relief where all food desires could be satisfied: “*I tend to eat what I can’t eat at home, so always unhealthy*” (FG1_F21); “*when I’m eating out "I might as well treat myself" and treat myself for nothing*” (FG4_F21); “*when I lived at home, I would always eat really healthy, so whenever I go out, I tend to eat a lot of junk food*” (FG1_F21). One participant’s personal experience confirmed that: “*usually when I go out with my friends or family, I eat just such trash food. And restaurant food to begin with is already so caloric, and then you just add on top of it, let’s get appetizers and desserts*” (FG4_F19).

High school had also a strong determinant on eating habits; most of the time, eating in secondary school was related with a negative experience: “*a lot of times in high school I just ate chips, because I just hated my school lunch, it was pretty bad. But I think if the school lunch is the only thing that’s available to you, it’s definitely going to affect what you’re eating and how you’re eating*” (FG4_F21). Several students reported that they did not feel that the school meal was healthy, due to limited choices. One remembered: “*we always used to joke about saying that pizza counted as a vegetable, we had to get a vegetable but pizza counted, so we’d always get pizza*” (FG4_M19). However, almost all of the participants agreed that nowadays, schools are getting more involved in providing healthy options than in the past: “*I think our school definitely they had healthier options*” (FG5_F19).

##### Friends and Media Pressure

Young adults are often influenced by their peers for many habits, and also when eating behaviors are involved [29]; as one male participant, who had a high frequency in activity level and played in a team, said: “*there is just so much social pressure to eat healthy around other people*” (FG3_M23). As one female student reported: “*I think every girl has this kind of thing and you have some pressure from your friends and if you will see them wearing beautiful dresses you want to lose weight or something*” (FG5_F24). Another explained: “*I think general rule of thumb, if you see people* [friends] *that look healthy, that we tend to ask someone, what do you eat? How do you do that?*” (FG5_M21). Usually, meals with friends tended to be not healthy: “*when I’m with my friends in the evening we do tend to eat heavier meals, which make me feel pretty sick the next morning*”. However, for someone else, the experience was the opposite: “*I think the big thing that changed for me was when I came here at Cornell, I saw other people and their eating habits, and some of them were eating lean or eating healthier, and I tried to pick up on some of those too*” (FG4_M21).

Many participants raised concerns about the role of television and other mass media on how an adolescent or young adult should look: “*I just feel like in the media, you see all these images of celebrities and their body type is glorified, so you just want to eat healthier to look like that*” (FG4_F19). In addition, they also considered advertisement on TVs for candies and other sweet foods to be negative communication on what to eat, as one participant said: *“…there’s all these ads on TVs for candies and stuff like that… kids would rather have the bright colors, the fun candies and stuff that aren’t necessarily healthy”* (FG1_F21).

#### 3.2.3. University Environment and Student Life 

Besides human physiology, the physical environment is also another element that can strongly shape our food choices [36]. In general the surroundings where you are living can strongly determine your diet: “*I also think like your environment that you’re in and that like you’re constantly in really affects how you eat*” (FG3_M19).

The university environment could have both a positive and negative influence on eating habits, as one participant explained: “*I think if the community is driven to be healthier, then I think once you’re in that environment, it tries to influence you to be healthier. And seeing other people around you eat healthy and want to be healthier is a big influencer on changing your habits. And vice versa*” (FG2_F20). For example, most of the students thought that the dining halls strongly influenced their eating habits. Some students started to eat irregularly when starting college: “*I eat irregularly, like sometimes for dinner I just don’t want anything in the dining halls and I’ll just eat cookies or the ice cream*” (FG5_F19); “*I probably eat more meat at college, I don’t know, just a lot of food*” (FG5_F19). When asked what events could make a person gain or lose lots of weight, someone said that going to college made people gain weight: “*having that sort of unrestricted freedom of being able to choose whatever you want to eat, and also having a meal plan where it’s like an “all-you-can eat” buffet*” (FG1_F20). One participant shared a personal experience and said: “*I need to go eat every meal at the dining hall. And once you’re at the dining hall, you have unlimited food, so I feel like I overate a lot in the dining halls. And now living off campus, I’m able to just buy what I want to cook, and sometimes I cook all my food at once. So I can plan, this is for lunch, this is for dinner. So I can do better with portion control*” (FG1_F21).

For some other students, especially athletes, having the dining hall always available and close to the dormitory or workplace was instead an advantage: “*it was good to have the dining halls right there so you could kind of eat whenever you wanted to. So it helped me stay healthy and had a good eating pattern for that kind of lifestyle. And then, I think once when I got off campus, it’s like harder to keep up with good eating patterns*” (FG6_M21). Student life could be a critical period regarding unhealthy changes in lifestyle behaviors: “*I also sometimes skip lunch when I have class or studying to do, and a lot of times when I’m studying I also eat junk food, try to keep myself awake*” (FG4_M21).

Table 4 summarizes the main barriers and enablers that are associated with health decisions during college life.

## 4. Discussion

Using an adapted version of an Ecological Model used by Deliens et al. [29], we developed a framework that included individual (intrapersonal), social (interpersonal), university environment (community settings), and students’ life factors as influences affecting eating habits. This model integrated individual healthy and unhealthy eating patterns, in combination with the main barriers and enablers that are associated with health decisions during college life. Many researchers [4,15,37,38,39] identified a great number of factors that may contribute to the malnutrition epidemic, and related health problems (e.g., weight gain and other dietary disorders) in emerging adulthood: unhealthy eating habits increased when young adults leave their home circumstances, such as lower consumption of healthy options (i.e., fruit and vegetables), irregular meals (e.g., breakfast skipping), and increasing intakes of unhealthy snacks and other “junk food” (e.g., fried food). For college students, the transition phase from living at home to living alone/with roommates during the period of postsecondary education, is one of the most important life changes, and many food choices are deeply involved in this change.

As indicated by other authors [2,3,4,35], the most common factors that are reported as barriers to a healthy diet are time constraints, the high price of food items, and their availability, followed by the lack of motivation in food preparation, which is strongly related to intention. Regarding the latter barrier, as reported by Menozzi, Sogari & Mora [35], intention is the main factor in predicting behavior regarding the consumption of healthy foods, such as fruits and vegetables. Therefore, we believe that nutrition professionals within the university community should design programs and tools that can help students to be more motivated in choosing healthy food. During the focus groups, students realized the strong role of college facilities in influencing their eating habits. In fact, when students start college, they will face a new (food) environment (e.g., all-you-can-eat formula dining), which can have strong impact on their eating habits and intention to perform a healthy behavior. Interventions across campus dining facilities should decrease the potential barriers to healthy food, and increase self-efficacy and behavioral controls, to encourage students to embrace a better diet [40].

Among the social enablers, students found that having the support of friends to be active in healthy eating was an important stimulus. We also observed that students who have a higher frequency of physical activity believe that social pressure helps them to stay healthy. Parents also have a crucial role, both positive and negative, in shaping the concept of healthy eating and in encouraging children in healthy activities, both related to eating (e.g., food preparation) or more physical (e.g., sport, outdoor activities). We noticed how perceived benefits of healthy eating also influence the intention to consume healthier food [41], which seems to be more easily achieved if students start planning their meals (self-control technique). Moreover, university characteristics, such as living arrangements (i.e., dormitory, off-campus, with parents) or academic schedules (e.g., classes, exams, etc.), also influence the relationships between individuals and their eating behaviors [18,29,42], and they should be taken into account when designing effective and tailored multilevel intervention programs.

Finally, it should be noted that some barriers for certain individuals, might be perceived as potential drivers by others. For instance, and not surprisingly, some students stated that “all-you-can-eat” formulas have a negative impact on the amount and quality of food consumed, whereas others believed that these types of dining halls facilitated their ability to have a healthy diet.

The focus groups confirmed that both lifestyle and behavioral factors are strongly associated with dietary patterns among college students: participants were aware that “being a healthy person” was not just exercising and eating healthy foods, but also taking time for yourself and being an overall happy individual.

One of the methodological limitations to the current study is that these results cannot be automatically generalized to the whole population of university students, when considering the specific and limited sample of participants (i.e., US college environment, healthy BMI status, other). Another limitation is related to the presence of students who might have been more interested in this topic, and decided to participate at the focus group, leading to “selection bias”.

## 5. Implications

More precision in the relationship between food and health is a topic of growing importance on the public agenda [43]. Nevertheless, even with wide recognition that the food that we consume has a strong impact on our health, consumers’ food preferences do not always lead to the best nutritional choices. A better understanding of the link between diet and health among college students is important for developing programs and behavioral change strategies to improve their lifestyle in general, and to reduce diet-related diseases in particular [9].

This study highlights the importance of consulting college students when developing healthy eating interventions across the campus for dining services or programs. As suggested by Stok et al., [10], researchers in the food and nutrition field should not only focus on individual-level factors, but they should also integrate socio-ecological aspects into the analysis. Dining halls and other University facilities should ensure the availability of healthy food choices, as well as promoting physical activity practices regularly. They should also provide food education and food preparation classes, to make students more knowledgeable on how to cook and better plan meals.

Giving college students the necessary skills to be more aware of what a healthy diet style means would empower them to make better food choices throughout their life. As suggested by many authors [4,44], interventions should be specific for the targeted population (i.e., young adults) in order to help individuals to behave accordingly with their healthy intentions. For instance, social media facilitates the interaction between individuals and organizations (e.g., university administrators and food researchers), in order to provide tailor-made information [29,45]. This aspect can be helpful in promoting healthy diets without creating eating disorders. In addition, price reductions for high-cost foods in campus facilities, such as dining halls and cafeterias, should also facilitate the purchase of more healthy options (e.g., fruits and vegetables). Environmental modifications can include changing and/or labeling healthy food options to make them more appealing, while creating a point of nutrition information where students can see healthy food options.

## 6. Conclusions

The aim of this study was to identify factors driving healthy lifestyle behaviors among US college students. Opinions and recommendations for effective and tailored-made intervention programs or environmental modifications that support healthy eating were presented, using an ecological framework that combined psychological, social, and environmental strategies.

Consumer behavior scientists typically do not contribute to the scientific debate about what is best to eat from a nutritional point of view or give recommendations about dietary components for the specific amounts and limits for food groups. In this study, we instead tried to understand the individual, social, and environmental factors that influenced students’ healthy eating choices. Our results suggest that participants were influenced by individual, social, and university environmental factors.

The Ecological Model can help university communities to gain more insights into how and why students make certain food choices, and support them in staying healthy.

Colleges and dining halls on campuses should acknowledge their crucial role in guiding healthy eating behaviors, and be the first subjects to be interested in creating a healthy environment for the students. Unless they start understanding the reasons behind unhealthy eating behaviors of young adults, effective policies and managerial strategies to fight malnutrition (obesity, anorexia, micro-deficiency) cannot be developed.

The next step of this research will include the collection of a larger and more representative sample size, especially when taking into consideration the socio-cultural differences of college students between the US and other Western countries. Considering that the same negative trend of overweightness and unhealthy eating behavior among children, adolescents, and young adults is emerging in Europe, and also in Mediterranean countries [46], discussions on potential and future studies addressing this problem in a national context are advised. In addition, further research should evaluate whether specific tailor-made interventions are effective in changing behaviors towards a healthy lifestyle.

## Figures and Tables

**Table 1:** Short version of the Focus Group questions guide.

Question Type	Questions
Opening and warm-up questions	Presentation of the research topic and participants (demographic characteristics and some general eating habits like “what do you have for breakfast?)
Introduction/Projective techniques	Could you list five habits related to healthy and unhealthy eating? Could you mention the first types of food/food products you consider healthy?
Transition questions (to move into and between key questions)	How do you think the concept of healthy eating has changed? Were you involved in cooking preparations in the past? What changes happened in your cooking habits since you started college?
Main key questions	What different eating behaviors do you have between eating out and at home? What is for you the meanings of the word “healthy” and “unhealthy”? What is your eating behavior to stay healthy? What are the consequences of having a healthy eating behavior? How may have the community (e.g., colleges) impacted on your healthy and unhealthy habits? How can a parent/guardian positively/negatively influence on children’s eating behavior?
Projective technique (i.e., showing images of overweight/underweight adults/children)	What comes into your mind (e.g., thoughts) when you see these images on obesity, overweightness, and a healthy body weight?
Ending	Are there any other opinions related to the topic? Is there anything else you would like to share?

**Table 2:** Characteristics of focus group participants (*n* = 35).

Group Characteristics	Responses	%	Mean ± SD
Race/ethnicity	White/Caucasian Asian (excluding South Asian) African American South Asian	80 11 6 3	
Gender (female)		66	
Age (years)			20.4 ± 1.5
Body Mass Index (BMI)			23.2 ± 4.5
Field of study	Business Scientific Humanistic Info not provided	42.9 34.3 14.3 8.5	
Students with an extra job	Job No job Info not provided	42.9 40 17.1	
Physical activity			
	No exercise	15.2	
	Exercise 1 time per week	21.2	
	Exercise 3 times per week	42.4	
	Exercise 5 times per week	21.2	
Self-assessment weight status			
	Underweight	9.1	
	Normal weight	66.7	
	Overweight	21.2	
Population Area (size of the city)			
	<5000 inhabitants	15.1	
	Between 5000 and 50,000 inhabitants	27.3	
	>50,000 inhabitants	57.6	

**Table 3:** Top 12 self-reported healthy and unhealthy eating habits of the participants.

Healthy Eating Habits	Frequency (*n*)	Unhealthy Eating Habits	Frequency (*n*)
Consumption of fruit and vegetables	26	Irregular meals	25
Drinking water	13	Sweet food (i.e., dessert, ice-cream, candy, chocolate)	21
Balanced diet	12	Unhealthy snacks	15
Portion control	8	High salty and fat food (i.e., fried food) intake	13
Having breakfast	8	Overeating	10
No sweet food	8	Skipping breakfast	10
No oils/fat (e.g., less sauces)	7	Over protein consumption (i.e., too much meat, eggs)	5
No processed food (i.e., whole food)	7	Eating disorders	5
Regular meals	7	Low water consumption	5
Protein consumption	7	Drinking soda	4
Self-prepared meals	6	Low fruit and vegetable consumption	4
Healthy snack (i.e., nuts)	5	Coffee consumption	3
Other		Other	

Notes: “Other”: eating habits that have been mentioned only one or two times. The researchers decided not to report them.

**Table 4:** Summary of the main barriers and enablers to a healthy diet among college students (*n* = 35).

BARRIERS	ENABLERS
**Individual-level** Not exercising Not eating healthful food Time constraints Unhealthy snacking Convenience food Bad mood & stress High prices Junk food home availability	**Individual-level** Maintenance of healthy lifestyle Healthy eating habits Food knowledge and education Meal planning Involvement in food preparation Physical activity Being portion-aware
**Social-level** Parental food behavior and influence Friends pressure and influence Low food culture	**Social-level** Friends pressure and influence Parental food behavior and influence
**University Environment** College’s dining services Availability of high-calorie food and fast food	**Environmental-level** College’s dining services

Source: own elaboration.
